# *Notaulax yamasui* sp. n. (Annelida, Sabellidae) from Okinawa and Ogasawara, Japan, with notes on its ecology

**DOI:** 10.3897/zookeys.660.11228

**Published:** 2017-03-07

**Authors:** Eijiroh Nishi, João Gil, Katsuhiko Tanaka, Elena K. Kupriyanova

**Affiliations:** 1 College of Education and Human Sciences, Yokohama National University, Hodogaya Yokohama, 240-8501, Japan; 2 CEAB-CSIC, Carrer d’accés a la Cala Sant Francesc, 14, 17300 Blanes (Girona), Spain / CCMAR, Universidade do Algarve, Campus Gambelas, 8005-139 Faro, Portugal; 3 Department of Marine Biology, School of Marine Science and Technology, Tokai University, Orido, Shimizu, Shizuoka, 424-8610, Japan; 4 Marine Invertebrates, Australian Museum Research Institute, Australian Museum, 1 William Street, Sydney NSW 2010, Australia

**Keywords:** Boring species, coral reef, new species description, Polychaeta, taxonomy, worms

## Abstract

The polychaete *Notaulax yamasui* **sp. n.** (Sabellidae) is described from Okinawa and Ogasawara, south Japan, where it was found living embedded in a dead skeleton of the coral *Porites* sp. The new species is characterized by the presence of a pigmented sub-distal swelling on the tips of the crown radioles, a unique feature among species of the genus. Besides, its collar chaetae have an L-shape orientation, and the dorsal basal flanges of the branchial lobes are long and have a dorsal joint.

## Introduction

A revision of the Japanese sabellid polychaetes belonging to the genera *Megalomma* Johansson, 1925, *Notaulax* Tauber, 1879, *Parasabella* Bush, 1905 and *Sabella* Linnaeus, 1767, is in progress. In the course of this revision, several Japanese collections are being revised for specimens belonging to these genera. As a result, two specimens belonging to the same species showed radioles with sub-distal swellings, like those found in *Sabella discifera* Grube, 1874 and in *Bispira brunnea* (Treadwell, 1917), as reported by Tovar-Hernández and Pineda-Vera (2008). These swellings can be pigmented, in which case they superficially resemble the compound eyes of *Megalomma* and *Stylomma* Knight-Jones, 1997. Other main features of the specimens include the long flanged radiolar lobes (similar to those in *Notaulax*, *Stylomma*, and *Anamobaea* Krøyer, 1856), and simple radiolar eyes (like those in *Notaulax*, *Anamobaea*, and *Hypsicomus* Grube, 1870). All these genera were revised or described by Rullier and Amoureux (1970), Perkins (1984), Knight-Jones (1997), Knight-Jones and Perkins (1998), Fitzhugh (2002) and Capa (2007). Further information on these genera can also be found in Fitzhugh (1989, 2003) and Capa et al. (2014).

The specimens collected at Okinawa and Ogasawara (south-western Japan) were studied using both light and scanning electron microscopy (SEM) for their external morphology, and through histological cross sections at different levels of the radioles for the internal anatomy of the radioles and their sub-distal swellings. As a result, the specimens were determined to belong to an unknown species of *Notaulax*, which is described below as a new taxon.

## Material and methods

The specimens were collected together with the surrounding coral at shallow water by hand, using chisels to break pieces of the coral, and fixed in the laboratory with a 10% seawater-buffered formalin solution. Some parapodia were removed from the body and prepared for microscopy observations. For light microscopy observations the parapodia were placed on a microscope slide, covered with a cover slip, and gentle pressure was applied in order to observe the chaetae and uncini. Histological sections were made from radioles embedded in paraffin, cut on a microtome, and stained with Sudan Black B. For SEM observations, the parapodia were run through a series of increasing concentrations of ethanol (80, 90, 95, 99 and 100%), air-dried, coated with palladium and platinum, and viewed in a Hitachi S-800 SEM. The holotype and paratype were deposited in the Coastal Branch of Natural History Museum and Institute, Chiba at Katsuura, Chiba, Japan (catalogue code, CMNH-ZW). The terminology for the anatomical structures of *Notaulax* follows Fitzhugh (1989, 2002).

## Systematics

### Genus *Notaulax* Tauber, 1879

#### Notaulax yamasui sp. n.

Taxon classificationAnimaliaORDOFAMILIA

http://zoobank.org/841FE0ED-E2E5-44E7-AFB4-8FDB399251D9

**Figure 1.**
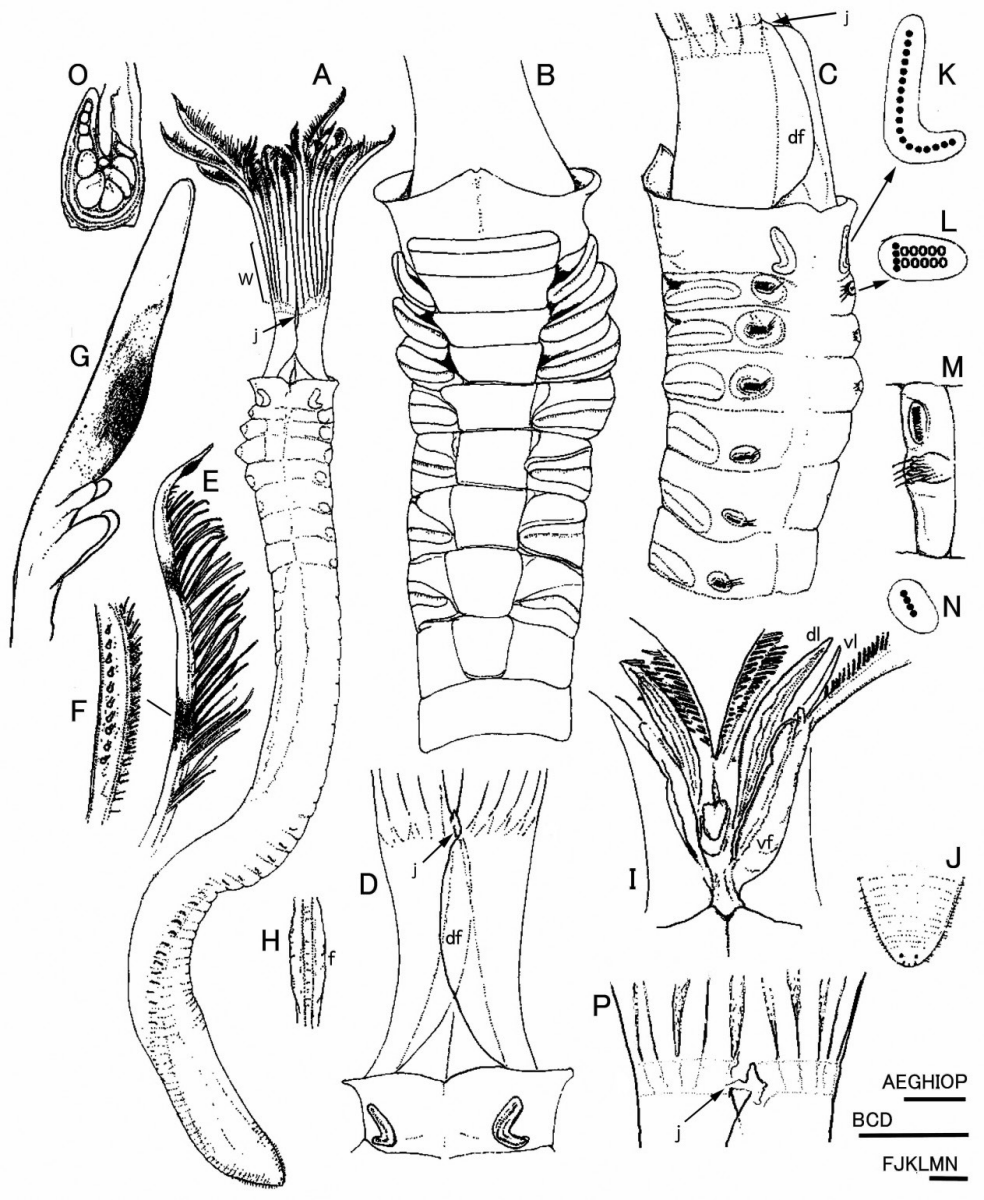
*Notaulax yamasui* sp. n.: **A** holotype, dorsal view **B** ventral view of thorax **C** latero-dorsal view of left side of thorax **D** dorsal view of first chaetiger and radiolar base **E** distal side view of radiole **F** middle region of radiole showing a row of simple radiolar eyes **G** lateral close up view of tip of radiole, with pigmented sub-distal swelling **H** basal part of radiole showing paired longitudinal flanges (f) **I** schematic ventral view of interior of crown showing dorsal lip (dl), ventral lip (vl), ventral flange of radiolar base margin (vf) **J** posterior abdomen and pygidium, showing eye-spots **K** schematic arrangement of collar chaetae, right side **L** schematic arrangement of thoracic chaetae from second chaetiger, black spots representing superior chaetae, white circles representing inferior chaetae **M** anterior abdominal segment, left side view **N** schematic arrangement of neuropodial abdominal chaetae **O** cross-section of radiole, middle region **P** base of radioles and inter-radiolar membrane. Abbreviations: df, dorsal basal flange; dl, dorsal lip; f, longitudinal flange; j, junction of dorsal basal flange; vf, ventral flange; vl, ventral lip; w, inter-radiolar membrane. Scale bars 1 mm (**A, B, C**), 0.5 mm (**D, J**), 0.25 mm (**E, F, H, K, P**), 0.1 mm (**G, I, L, M, N, O**).

**Figure 2.**
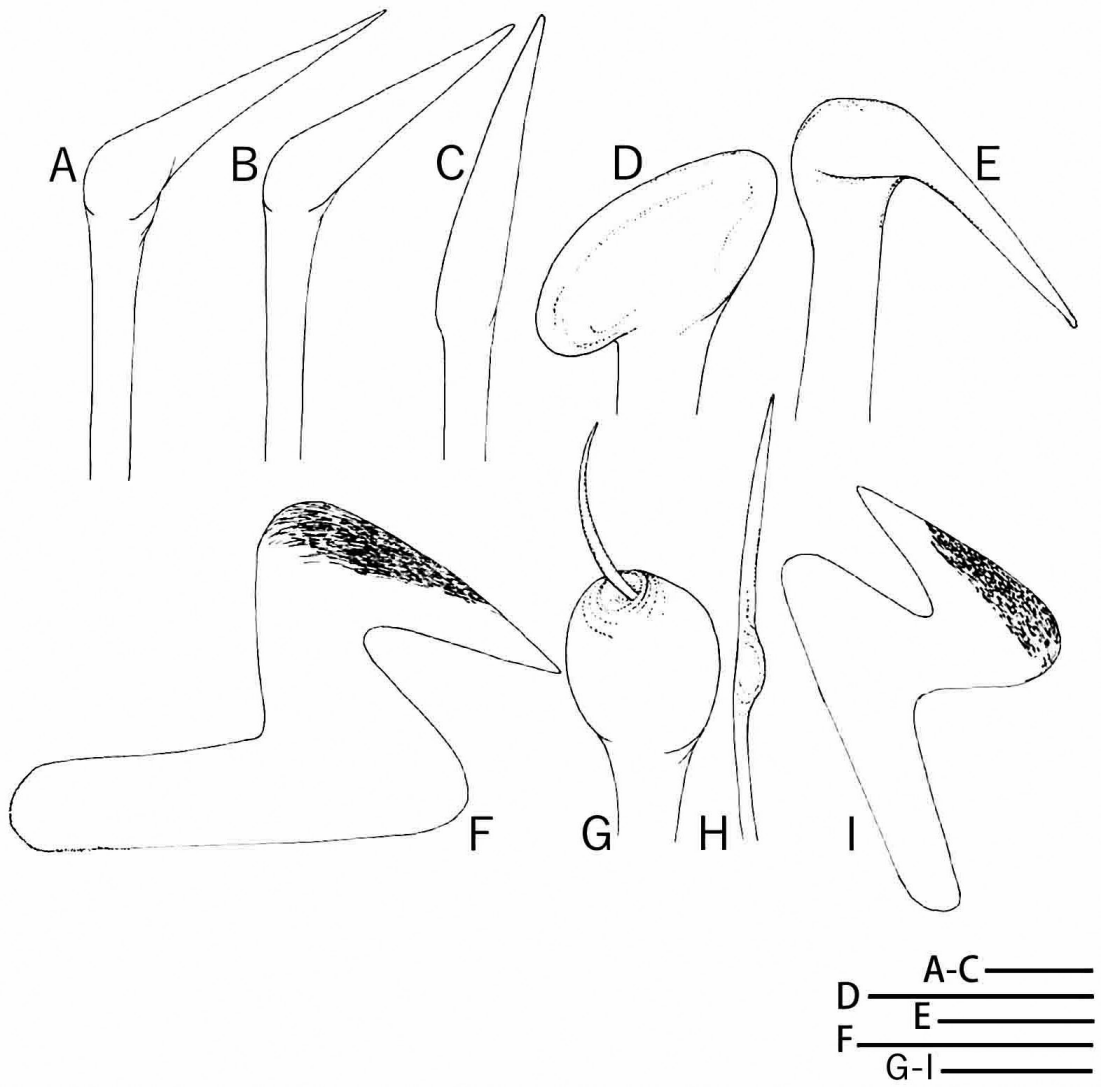
*Notaulax yamasui* sp. n. Chaetae of thorax (**A–E**) and abdomen (**G, H**) drawn from SEM micrographs, and uncini (**F, I**), drawn under a dissecting light microscope. **A–B** collar chaetae **C** superior thoracic chaeta **D** inferior thoracic chaeta **E** companion chaeta, dorsal view **F** thoracic uncini **G** inferior abdominal chaeta, anterior abdominal chaetiger **H** inferior abdominal chaeta, posterior abdominal chaetiger **I** abdominal uncini. Scale bars 20 µm (**A–C**), 50μm (**D**), and 30μm (**E–I**).

**Figure 3.**
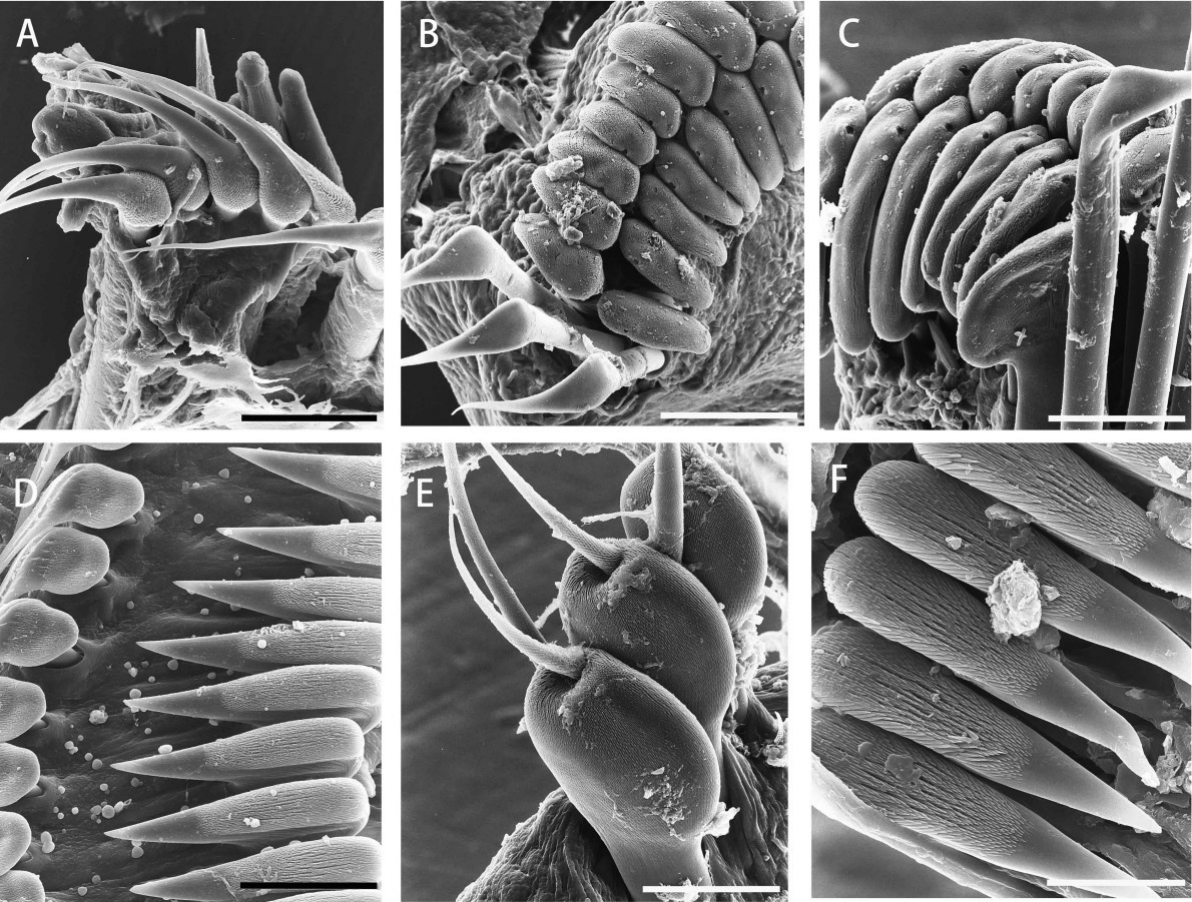
*Notaulax yamasui* sp. n. SEM micrographs of chaetae and uncini. **A** collar chaetae **B** 3^rd^ left notopodial thoracic fascicle **C** detail of B, showing superior chaetae **D** uncini and companion chaetae, 3^rd^ thoracic neuropodial torus **E** inferior abdominal spatulate chaetae showing distal mucros from 7^th^ fascicle of abdomen, lateral view **F** abdominal uncini. Scale bars 30μm (**A**), 60μm (**B**), 40μm (**C**), 30μm (**D, E**), 12μm (**F**).

**Figure 4.**
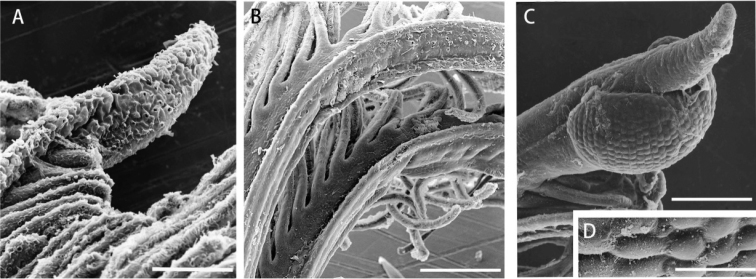
*Notaulax yamasui* sp. n. (**A, B**) and *Megalomma* sp. (**C, D**), SEM micrographs of anterior and middle parts of radiole and distal tip with a distal swelling in *N. yamasui* sp. n. and with a compound eye in *Megalomma* sp. **A** close-up view of a sub-distal radiolar swelling **B** middle part of radiole showing pinnules and dorsal flange **C** compound eye on radiole **D** close-up view of surface of compound eye. Scale bars 75 µm (**A**), 200 µm (**B**), 300 µm (**C**), and 30 µm (**D**).

**Figure 5.**
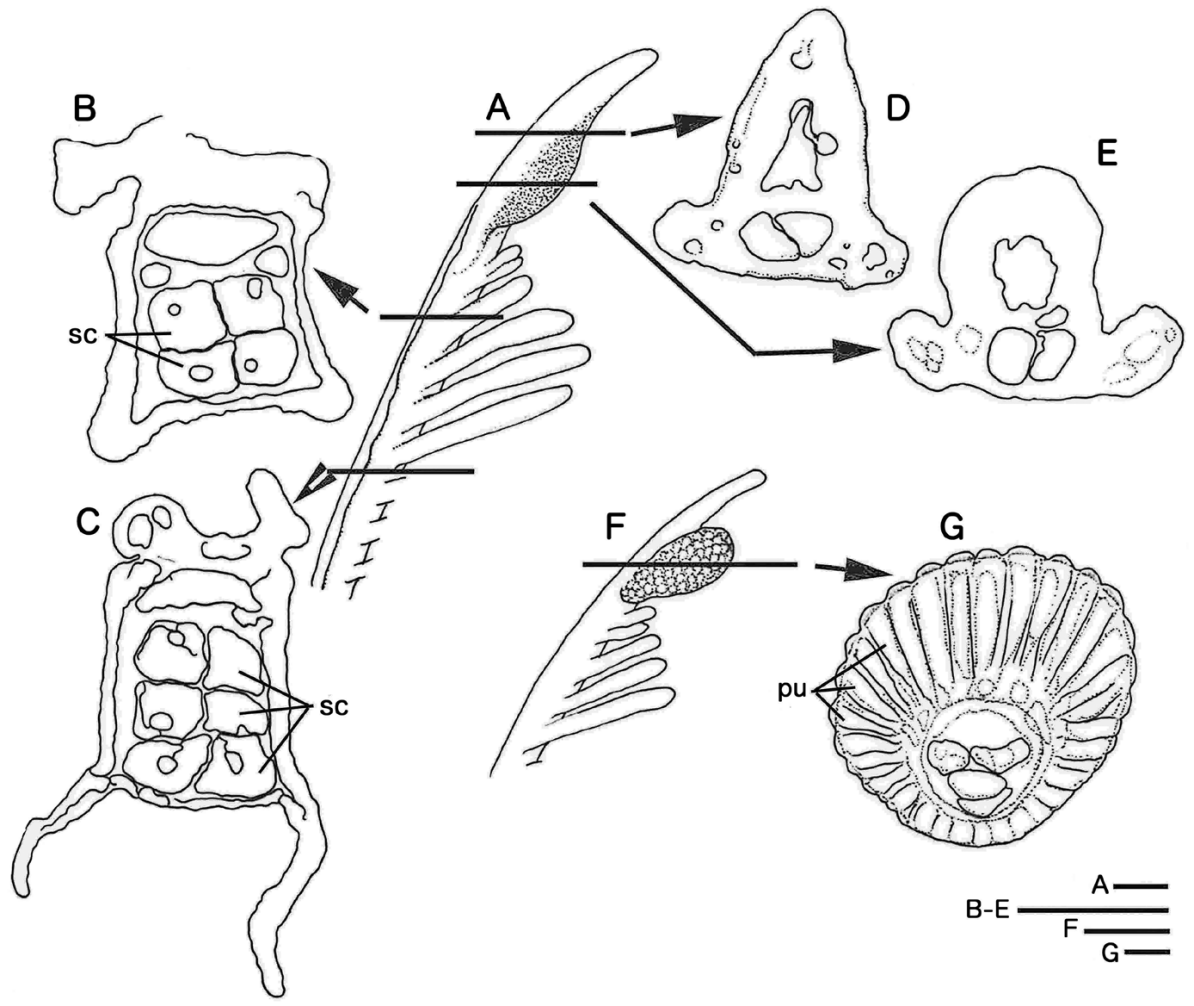
*Notaulax yamasui* sp. n. (**A–E**) and *Megalomma* sp. (**F, G**), internal structure of radiole. *Notaulax yamasui* sp. n. **A** radiole tip, lateral view **B, C** internal structure of proximal region of radiole **D, E**, internal structure of sub-distal swelling. *Megalomma* sp. **F, G** internal structure of compound eye **F** radiole with compound eye, lateral view **G** internal structure of compound eye, with many individual photoreceptor units (pu) **B–E** and **G** are drawn from sliced sections of eyes and radioles. Abbreviations: sc, skeletal cells; pu, photoreceptor unit. Scale bars 100μm (**A**), 200μm (**B–E**), 300μm (**F**), 20μm (**G**).

Figs 1 , 2 , 3 , 4A–B , 5A–E

##### Material examined.

Holotype: CMNH-ZW00217, complete specimen with fragment of tube, extracted from living coral mass of *Porites* sp., collected in the subtidal zone (0–2 m) of a shallow coral reef area at Maeda-Misaki Cape, 26°26.716'N, 127°46.329'E, Okinawa Island, Ryukyu Archipelago, south-western Japan, Pacific Ocean, 13 February 1996, by hand, coll. by E. Nishi. Paratype: CMNH-ZW00220, incomplete specimen lacking posterior abdomen and tube, collected on a dead *Porites* sp. coral colony, at Kominato, Chichi-jima Island, Ogasawara Archipelago, south-east Japan, Pacific Ocean, 16 July 1999, coll. by Prec. Institute Co Ltd.

##### Comparative material.

*Megalomma* sp., CMNH-ZW uncatalogued, Yoshio, Katsuura, Boso Peninsula, Japan, subtidal, coll. by E. Nishi.

##### Diagnosis.

Pigmented sub-distal swelling on tips of crown radioles; collar chaetal row in L-shape orientation; dorsal basal flanges of radiolar lobes long and with a dorsal joint.

##### Description.

*Tube* dark brown, thin and membranous. Body and radiolar crown pale in preserved specimens, except for light brown collar and for two (upper and lower) brown bands on distal free region of radioles (Fig. 1A, E).

*Body* of holotype 40 mm long (excluding crown) for 130 chaetigers (including thorax and abdomen); thorax 4 mm long and 2.0-2.5 mm wide, excluding chaetae; radiolar crown 6 mm long, radiolar lobes 1 mm long. Paratype similar in size, body 6 mm long (posterior portion of abdomen missing) for 32 chaetigers, thorax 3 mm long and 1.5 mm wide, excluding chaetae; radiolar crown 7 mm long, radiolar lobes 1.5 mm long.

*Crown* with 16 pairs of radioles, joined by inter-radiolar membrane (Fig. 1C, D, P), inter-radiolar membrane about 1/2 length of radiole length (Fig. 1A); radiolar lobes with narrow dorsal flanges (Fig. 1 A, C), flanges closed at lower level of inter-radiolar membrane by dorsal joint (Fig. 1P), and free proximally (Fig. 1C, D); ventral margins of radiolar lobes also flanged, ventral flanges free (Fig. 1I); radioles with 4 or more skeletal cells in cross-section (Figs 1O, 5B, C, D, E), with paired longitudinal flanges on outer surface, more prominent at basal region near inter-radiolar membrane (Figs 1H, 4B, 5C), turning distally into flattened long tongue-shaped tips (Figs 1A, E, G, 4A, 5A); each radiole with one pigmented sub-distal swelling on inner side (not pigmented in paratype, showing same color to rest of body) (Figs 1A, E, G, 4A, 5A) and 8-12 pale brown simple radiolar eyes in single row on each side, at lateral margin of central region of radioles (within lower brown band) (Fig. 1E, F). Dorsal lips long, tapered to slender, with supporting mid-rib, joined to adjacent radiole (= radiolar appendage), but not to basal pinnule (Fig. 1I). Ventral lips tapered and small, merging proximally into parallel lamellae (Fig. 1I); ventral sacs absent.

*Thorax* with eight chaetigers; posterior peristomial ring collar entire, without dorsal or ventral slits, well separated from peristomium, with straight brown line above ventral glandular shield (Fig. 1B), mid-dorsal margin slightly embayed, lateral margin transverse to body axis and extending well above junction of radiolar crown with thorax, ventral margin raised in middle and incised ventrally with small notch on midline (Fig. 1B, C, D). First ventral glandular shield rectangular, divided transversally, with nearly straight anterior margin, slightly wider than shield of chaetiger 2 and about 2/3 longer (Fig. 1B). Other thoracic ventral glandular shields sub-trapezoidal (broader anteriorly), margins postero-laterally indented by tori. Abdomen with 122 (holotype) and 24 (paratype, posterior region missing) chaetigers. Pygidial eyespots present (Fig. 1A, J).

*Collar chaetae* spine-like, each with knee wider than shaft (Figs 2A, B, 3A), in longitudinal rows, curved outwards posteriorly (Fig. 1C, D, K). Superior chaetae of thoracic notopodial fascicles spine-like, similar to chaetae in chaetiger 1 (Figs 2C, 3B, C) and in short row (Fig. 1L), dorsal to paleate inferior thoracic notochaetae with hoods distally rounded (Figs 2D, 3B, C), arranged in two transverse rows (Fig. 1L). Thoracic neuropodial fascicles with avicular uncini, with several minute teeth above main fang, prominent breast and handle longer than distance between breast and main fang (Figs 2F, 3D). Companion neurochaetae in row parallel and anterior to uncini, with broad, thin teardrop-shaped blades at right angle to shafts, pointing anteriorly (Figs 2E, 3D). Abdominal neuropodia with neuropodial fascicles of paleate chaetae in short transverse rows (Fig. 1M, N); paleate neurochaetae with distal mucros shorter than hooded area in anterior abdominal segments (Figs 2G, 3E), mucros becoming longer than hooded area in posterior abdominal segments. Paleate neurochaetae numbering 4 per fascicle on most anterior abdominal segments (1st to 7th), 3 on median segments (8th to 20th), and one or two on posterior chaetigers. Superior neuropodial abdominal chaetae slender and straight, with or without sub-distal bulge (Fig. 2H), one per fascicle on anterior abdominal chaetigers (1st to 20th) and two to three in posterior ones. Abdominal notopodial avicular uncini similar to thoracic uncini (Figs 2I, 3F).

##### Habitat.

*Notaulax yamasui* sp. n. is known to live in the subtidal zone, embedded in dead coral masses of *Porites* sp.

##### Etymology.

The new species is named after Dr. Terufumi Yamasu, Emeritus Professor of the University of the Ryukyus, Japan, for his great contribution to the development of the Okinawan marine biology.

## Discussion

### Systematics

Under the stereo-microscope the radiolar sub-distal swellings of *Notaulax yamasui* sp. n., pigmented in the holotype, superficially resemble the typical radiolar compound eyes of the genera *Megalomma* and *Stylomma*, while other characters are typical of other sabellid genera lacking such eyes: the linear collar chaetae fascicles of *Notaulax*, *Panousea* Rullier and Amoureux, 1970, or *Panoumethus* Fitzhugh, 2002; the loosely aligned simple radiolar eyes of *Hypsicomus*, *Notaulax*, and *Anamobaea*; the long radiolar lobes of *Stylomma*, *Notaulax*, and *Anamobaea*. From these, *Panousea* and *Panoumethus* were ruled out from the beginning due to the presence of thoracic acicular uncini.

The fan-worm eyes and other photoreceptors are summarized in Bok and Nilsson (2016) and Bok et al. (2016). The compound eyes of *Stylomma* are stalked, which occurs neither in *Megalomma*, nor in the swellings of *N. yamasui* sp. n. The radiolar sub-distal swellings of the specimens of *N. yamasui* sp. n. were compared with the compound eyes of an unidentified *Megalomma* specimen collected at Katsuura, Chiba (Honshu, Japan). Scanning electron micrographs of *Megalomma* sp. eyes showed a surface structure analogous to the insect compound eyes, with many individual lenses arranged in a geometrical array (Fig. 4C, D). This does not occur in the sub-distal radiolar swellings of *N. yamasui* sp. n., where the surface of the swellings does not show any kind of special array (Fig. 4A). Moreover, while the former eyes have clearly defined edges, the latter have diffused edges around the swelling.

The internal morphology of both structures in *Megalomma* sp. and *N. yamasui* sp. n. compared through histological cross-sections showed ultrastructural differences: *Megalomma* sp. presents lenticular photoreceptor units (Fig. 5G), while the swellings of *N. yamasui* sp. n. are structurally similar to other regions of the radioles (Fig. 5B–E). These differences show that the new species lacks the compound eyes typical of *Megalomma* or *Stylomma*.

The remaining three genera (*Notaulax*, *Anamobaea*, and *Hypsicomus*) belong to a well-defined group inside the Sabellidae (Fitzhugh 1989: Clade IV in fig. 28; Nogueira et al. 2010: clade in figs 18–20, 22). These three genera share a number of features, including the presence of scattered simple radiolar eyes along the lateral margins of the radioles (Fig. 1E, F). However, *Hypsicomus* and *Anamobaea* can be easily separated from *Notaulax* and the new species by having the collar chaetae arranged in a bundle, instead of a long row. Besides, the spine-like shape of the superior thoracic notochaetae of the new species is typical of *Notaulax*, while in both *Hypsicomus* and *Anamobaea* thoracic notochaetae are elongated and narrowly hooded.

Finally, other characters typical for the genus *Notaulax* and also present in the new species, such as long flanged radiolar lobes, gave further support to its identification as a member of the genus. The genera *Hypsicomus* and *Notaulax* were partially revised by Perkins (1984) who, after examining the type species of *Hypsicomus*, the Adriatic *H. stichophthalmos* (Grube, 1863), redefined the genus and transferred to *Notaulax* all but the type species previously included in *Hypsicomus*. This means that the literature records of coral-boring *Hypsicomus phaeotaenia* *sensu lato* or *Hypsicomus* ssp. would be referable to *Notaulax* species (see below).

Among the members of the genus *Notaulax* (see Perkins 1984, Capa and Murray 2015), *N. yamasui* sp. n. is unique in having radiolar sub-distal swellings and L-shaped distributed collar chaetae. Another remarkable character of *Notaulax yamasui* sp. n. is the structure of the dorsal basal flange, which is rounded and long (Fig. 1A, D), with bases closed dorsally by a dorsal joint (Fig. 1P). A similar structure was reported in *Stylomma palmatum* (Quatrefages, 1866) by Capa (2008). In Japanese waters, the only recorded *Notaulax* species is *N. lyra* (Moore and Bush, 1904). *Notaulax yamasui* sp. n. is differentiated from *N. lyra* by the presence of radiolar subdistal swellings, a much longer inter-radiolar membrane which is about half the length of the radioles (Fig. 1A), and the color pattern of radioles (two or three brown bands in the former species, and reddish brown eyes pots occupying the basal three-tenth of radiole in the latter species) (Imajima and Hartman 1964).

The entire posterior peristomial ring collar is also an uncommon feature among *Notaulax* species, being described only in two other species: *Notaulax pyrrhogaster* (Grube, 1878) from Philippine Islands, and *N. alticollis* (Grube, 1868) from the Red Sea. Like in these two species, *N. yamasui* sp. n. also shows the ventral margin of the collar more or less extended forward, forming a triangular lobe. However, neither of those two species has radiolar distal swellings, nor the collar chaetae in an L-shaped arrangement. Besides, *N. pyrrhogaster* does not show simple radiolar eyes (likely not faded by alcohol, as according to Wiktor (1980), the syntype has been preserved in formalin), and *N. alticollis* has the group of radiolar eyes positioned along two rows with less than 15 eyes in each, corresponding to about 7 pinnules in length. *Notaulax yamasui* sp. n. has the radiolar eyes in a group corresponding to about 11-12 pinnules in length, with 8–12 eyes in a single row. Capa and Murray (2015) recorded *Notaulax* sp., having radiolar eyes (noted as radiolar ocelli) arranged in a single row or in teardrop-shaped groups. Other types of radiolar eyes and further details about their structure can be found in Bok et al. (2016).

### Ecology of *Notaulax*

The two types of *Notaulax yamasui* sp. n. were found living embedded in dead masses of coral *Porites* sp. Boring by worms in coral reefs is a common and very well-known phenomenon described as early as in 1902 by Gardiner (1902), and recently revised by Hutchings (2008). With the prevalent predation pressures at shallow coral reefs being high, the advantage of burrowing for protection into hard surfaces such as corals seems obvious, with positions submitted to currents and vertical surfaces being particularly favored by filter feeders to maximize feeding benefits and avoid sedimentation (Elias 1986, Hutchings 1986). Normally worms only bore into dead corals, or in the dead edges of living corals, avoiding contact with the soft parts. The recruitment by the worms is believed to be entirely *via* larvae or juveniles settling on the surface; as coral polyps are carnivores, the successful recruitment and subsequent boring is restricted mainly to the coral areas where polyps are damaged or very scarce (Hutchings and Murray 1982, Hutchings 2008).

Boring by worms plays an important role in the bio-erosion of coral reefs, but much less so than grazing by echinoids and fish, with boring polychaete species belonging to several families (the most important being Eunicidae, Lumbrineridae, Dorvilleidae, Oenonidae, Spionidae, Cirratulidae, and Sabellidae) and also Sipuncula (Warme 1975, Hutchings 1986, Hutchings and Peyrot-Clausade 2002, Hutchings 2008). Boring mechanisms in polychaetes can include mechanical (Eunicida) or chemical methods (Spionidae, Sabellidae, and probably Cirratulidae) (Hutchings 2008), and normally tubes or holes made by boring organisms can be recognized by their nearly constant diameter, as they are bored continuously to accommodate the growth of the host corals (Nishi and Nishihira 1999).

Many (if not all) Sabellidae *sensu* Kupriyanova and Rouse, 2008 secrete mucus tubes by ventral sacs, general body walls, ventral gland shields, and parapodial glands, and at least in five genera (*Sabella*, *Myxicola* Koch in Renier, 1847, *Pseudopotamilla* Bush, 1905, *Perkinsiana* Knight-Jones, 1983, and *Sabellastarte* Krøyer, 1856) the tubes are made of acid mucopolysaccharide-protein complexes (Chungtai and Knight-Jones 1988, Hutchings 2008). Hartman (1954) already suggested that the penetrating effect of *Notaulax* sp. (as *Hypsicomus phaeotaenia*) could be a result of a chemical action on the coral surface (see below).

Similarly, larvae of *Notaulax* species settle on dead corals, probably benefiting from the rugose surface for protection, while burrowing holes into the dead coral mass. A transverse section of a *Notaulax* sp. burrow in a *Porites* sp. coral is represented in Nishi and Nishihira (1999).

Many sabellids are known to live in hard carbonate substrates and some of them have been described as having their tubes embedded into substrates such as rocks (*Sabellastarte magnifica* (Shaw, 1800); *Pseudopotamilla reniformis* (Bruguière, 1789); *Parasabella saxicola* (Grube, 1861), as *Demonax brachychona* (Claparède, 1870); *Potamethus mucronatus* (Moore, 1923)), concretions of coralline algae (*Demonax langerhansi* Knight-Jones, 1983), shells or limestone (*Perkinsiana rubra* (Langerhans, 1880)), abalone shells (*Terebrasabella heterouncinata* Fitzhugh and Rouse, 1999) or shells of freshwater mollusks (genus *Caobangia*) (Jones 1974, Chughtai and Knight-Jones 1988, Fitzhugh and Rouse 1999, Kuris and Culver 1999, Simon et al. 2005, Moreno et al. 2006).

At least seven species of *Notaulax* live embedded in dead corals (see below), and the same is true for one undescribed *Megalomma* species (Chughtai and Knight-Jones 1988), one undescribed species of Fabriciidae (Hutchings and Peyrot-Clausade 2002), *Potamilla ehlersi* Gravier, 1906, *Megalomma claparedii* (Gravier, 1906) (as *Branchiomma*), *M. circumspectum*, Branchiomma cf. bairdi, *Megalomma mushaense* (Gravier, 1906) (as *Branchiomma mushaensis*), *Megalomma miyukiae* Nishi, 1998, *Perkinsiana fonticula* (Hoagland, 1919) (as *Parasabella*), *Amphicorina schlenzae* Nogueira and Amaral, 2000, *A. bichaeta* Capa and López, 2004, *A. perkinsi* Capa and López, 2004, *Amphiglena jimenezi* Capa and López, 2004, *Pseudobranchiomma minima* Nogueira and Knight-Jones, 2002, *Bispira paraporifera* Tovar-Hernández & Salazar-Vallejo, 2006, *B. melanostigma* (Schmarda,1861), *Pseudopotamilla intermedia* Moore, 1905, or *Pseudopotamilla fitzhughi* Tovar-Hernández & Salazar-Vallejo, 2006 (Gravier 1906, Nishi 1998, Nogueira and Amaral 2000, Nogueira and Knight-Jones 2002, Capa and López 2004, Tovar-Hernández and Salazar-Vallejo 2006), but the list is probably much longer. In many cases, lack of ecological data on the described species hides the boring habitat of the worm, while in others it is not clear whether the worms were embedded in the hard carbonate substrates or just associated with them.

Scleractinian corals seem to constitute the preferred habitat of the genus *Notaulax*. From the 20 described species of *Notaulax* valid according to Perkins (1984), besides *N. yamasui* sp. n., six are known to bore into coral masses (*N. nudicollis* (Krøyer, 1856); *N. occidentalis* (Baird, 1865); *N. marenzelleri* (Gravier, 1906); *N. pigmentata* (Gravier, 1906); *N. midoculi* (Hoagland, 1919); and *N. bahamensis* Perkins, 1984), and one was found associated with a fossil reef (*N. longithoracalis* (Hartmann-Schröder, 1980)) (Gravier 1906, Perkins 1984, Capa and López 2004, Tovar-Hernández and Salazar-Vallejo 2006). Additionally, Capa and Murray (2015) reported *Notaulax* spp. 1, 2 and 3 from the coral reef of Lizard Island, Great Barrier Reef, Australia. The remainder of the species have been described with no information on the substrates where they were collected, but one indeterminable species (*Notaulax* sp., in Fitzhugh 2002) was found in muddy sand.

Giangrande and Licciano (2004: fig. 4g) reported species richness of the genus *Notaulax* along global latitudinal belts. It is clear that *Notaulax*, being absent from the polar regions, has a preferentially tropical distribution, with the most records occurring between 30°N and S, and a clear domain in the northern hemisphere. This asymmetrical distribution between the hemispheres is probably simply due to a ‘concentration effect’, a consequence of the higher number of specialists working in the northern hemisphere, where some of the most studied marine faunas of the world are also located (Giangrande and Licciano 2004).

The latitudinal distribution of *Notaulax* fits almost perfectly the global carbonate production, especially as aragonite (Buddemeier 1997: fig. 1; Wood 2001: fig. 1), and by extension, the location of the scleractinian coral reefs (composed mainly by aragonite), also up to about 30°N and S, beyond which coral reefs are usually absent. *Notaulax* species seem to be typically borers, mainly in corals, but also in other carbonate (apparently mainly in aragonite) substrates. Besides the above cited species, references to *Notaulax* specimens as coral borers are frequent in the literature on coral reef polychaetes, especially as unidentified *Hypsicomus* species (e.g., Hartman 1954, Marsden 1960, Peyrot-Clausade et al. 1992, Nishi 1997, Hutchings and Peyrot-Clausade 2002), as *H. elegans* (see Gibbs 1969), *H. phaeotaenia* (see Hutchings et al. 1992), or as *Notaulax* sp. (Nishi and Nishihira 1999, Capa and Murray 2015).

## Supplementary Material

XML Treatment for Notaulax yamasui
